# Intracellular mono-ADP-ribosyltransferases at the host–virus interphase

**DOI:** 10.1007/s00018-022-04290-6

**Published:** 2022-05-10

**Authors:** Bernhard Lüscher, Maud Verheirstraeten, Sarah Krieg, Patricia Korn

**Affiliations:** Institute of Biochemistry and Molecular Biology, Faculty of Medicine, RWTH Aachen University, Pauwelsstraße 30, 52074 Aachen, Germany

**Keywords:** ADP-ribosylation, Alphavirus, Chikungunya virus, Coronavirus, Macrodomain, MARylation, Hydrolase, Interferon, PARP, Pattern recognition receptors, Signaling

## Abstract

The innate immune system, the primary defense mechanism of higher organisms against pathogens including viruses, senses pathogen-associated molecular patterns (PAMPs). In response to PAMPs, interferons (IFNs) are produced, allowing the host to react swiftly to viral infection. In turn the expression of IFN-stimulated genes (ISGs) is induced. Their products disseminate the antiviral response. Among the ISGs conserved in many species are those encoding mono-ADP-ribosyltransferases (mono-ARTs). This prompts the question whether, and if so how, mono-ADP-ribosylation affects viral propagation. Emerging evidence demonstrates that some mono-ADP-ribosyltransferases function as PAMP receptors and modify both host and viral proteins relevant for viral replication. Support for mono-ADP-ribosylation in virus–host interaction stems from the findings that some viruses encode mono-ADP-ribosylhydrolases, which antagonize cellular mono-ARTs. We summarize and discuss the evidence linking mono-ADP-ribosylation and the enzymes relevant to catalyze this reversible modification with the innate immune response as part of the arms race between host and viruses.

## Introduction

**Fig. 1:**
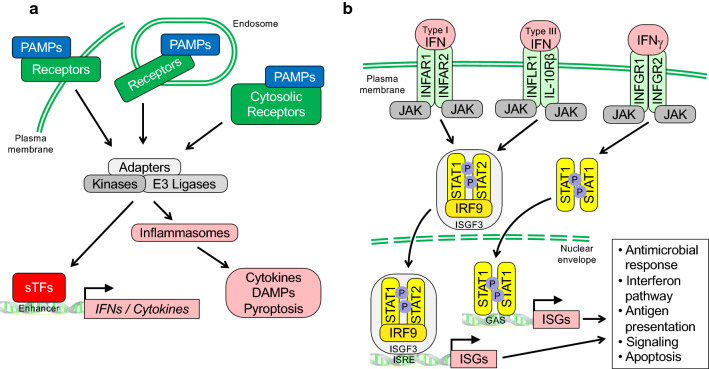
Schematic summary of signaling processes in innate immunity. **a** Pathogen-associated molecular patterns (PAMPs) serve as markers recognized by pattern recognition receptors (PRRs) that allow cells to distinguish between self and non-self. PRRs include membrane bound Toll-like receptors (TLRs) and cytosolic nucleotide-binding oligomerization domain (NOD)-like receptors (NLRs), retinoic acid-inducible gene I (RIG-I)-like receptors (RLRs), AIM2-like receptors (ALRs), and the cyclic guanosine monophosphate–adenosine monophosphate synthase (cGAS). These receptors read different PAMPs, which include conserved microbial components such as glycolipids, peptidoglycans, lipopolysaccharides, and various nucleic acids such as dsRNA and dsDNA, and stimulate signaling complexes that typically involve adaptor proteins and enzymes with kinase and ubiquitin E3 ligase activity. Subsequently, these activate sequence specific transcription factors (sTFs) such as IFN regulatory factors (IRFs) and NF-kB proteins, and inflammasomes. The latter are multimolecular complexes controlling proteolytic enzymes such as caspase-1, which activate IL-1 family cytokines [260, 261]. As a consequence, IFNs, pro-inflammatory cytokines and alarmins or DAMPs (damage associated molecular patterns) are released and disseminate potentially hazardous pathogen encounters. **b** Different interferons (IFN) interact with distinct heterodimeric receptors as indicated. Upon cytokine binding, Janus family kinases (JAKs) are stimulated that phosphorylate transcription factors of the signal transducer and activator of transcription (STAT) family. Complexes of STAT1 and STAT2 with IRF9 form the trimeric transcription factor ISGF3, which binds to IFN-specific response elements (ISREs). Dimeric STAT1 complexes recognize IFNγ activation sites (GAS). Both ISREs and GAS elements are commonly found in IFN stimulated genes

The innate immune system is our first line defense against pathogens. To mount a rapid response, it senses pathogen-associated molecular patterns (PAMPs) through pattern recognition receptors (PRRs) (Fig. 1a). These include membrane-bound toll-like receptors (TLRs) and cytosolic receptors such as retinoic acid-inducible gene I (RIG-I)-like receptors (RLRs), nucleotide-binding oligomerization domain (NOD)-like receptors (NLRs), AIM2-like receptors (ALRs), and cyclic guanosine monophosphate–adenosine monophosphate synthase (cGAS) and RNA helicases that sense nucleic acids [1–7].

PRRs recognize PAMPs to allow cells to distinguish between self and non-self. PAMPs consist of a variety of molecules with broad chemical properties, including conserved microbial components such as glycolipids, peptidoglycans, and lipopolysaccharides (LPS). Also, certain forms of RNA and DNA serve as PAMPs. Nucleic acid receptors sense distinct features of these polymers that are not present or not available in host cells. Examples are viral replication intermediates, double stranded (ds) RNA or dsDNA with a high non-methylated cytosine-phosphate-guanosine (CpG) content [8–10].

Upon interaction with PAMPs, PRRs induce signaling cascades that stimulate interferon (IFN) expression. Subsequently, these secreted cytokines induce the expression of IFN-stimulated genes (ISGs), which encode antiviral and antimicrobial factors and thus enable other cells to mount an antipathogen response [11, 12]. Some ISGs encode ADP-ribosyltransferase (ART) family members, in particular mono-ADP-ribosylating (MARylating) PARPs (Box 1) [13, 14]. In this review, we focus on the role of intracellular mono-ADP-ribosyltransferases in the antiviral response. This involves functions of ARTs as PAMP receptors and as regulators of both host and viral factors. Moreover, we summarize how viruses antagonize MARylation, which is achieved by ADP-ribosylhydrolases. These findings suggest that MARylation is an important arm of innate immunity and that the emerging mechanisms may offer opportunities to interfere with viral replication and/or immune modulation.

Of note, enzymes that poly-ADP-ribosylate (PARylate) substrates, such as PARP1, and extracellular mono-ARTs, referred to as ARTCs [14], have also been linked to innate and adaptive immunity. Discussing these findings in detail is beyond the scope of this review. We refer the reader to excellent, recent reviews that summarize and discuss these functions [15–17]. 

## Recognition of PAMPs and signaling by PRRs

TLRs are spanning either the plasma (e.g. TLR2, 4 and 5) or the endosomal membrane (e.g. TLR3, 7 and 8), recognizing a large variety of different PAMPs such as LPS by TLR4 and dsRNA by TLR3, and activate signal transduction pathways (Fig. 1a) [2, 18–20]. Cytosolic RLRs recognize PAMPs that include nucleic acids. For example, un-capped dsRNA promotes oligomerization of RIG-I and MDA5, while unmethylated CpG-rich DNA is sensed by cGAS, which then activates the adaptor mitochondrial antiviral signaling protein (MAVS) or stimulator of interferon genes (STING), respectively, to promote the assembly of signaling complexes. These contain different ubiquitin E3 ligases and kinases, which activate sequence specific transcription factors (sTFs) such as interferon regulatory factors (IRFs) 3 and 7 and NF-κB [5, 9, 21–27]. These sTFs stimulate the expression of genes encoding type I and III interferons (IFN) as well as pro-inflammatory cytokines in many different cell types [28–31]. In addition, the expression of IFNγ, the single type II IFN, is induced predominantly in certain immune cells, such as natural killer, innate-like lymphoid and T cells following PRR activation [32–34]. Together, these IFNs unfold signaling processes controlling the expression of genes whose products possess broad antiviral activities (Fig. 1b).

## Interferon regulated genes and their products

IFNs were originally identified due to their interfering antiviral activities [35, 36]. Once released, IFNs disseminate the information about preceding PAMP recognition and thus alert neighboring cells to potentially hazardous pathogens that were encountered [11]. Type I, II and III IFNs signal through IFNAR1/INFAR2, IFNGR1/IFNGR2 and IFNLR1/IL10Rβ heterodimers, respectively, and activate JAK-STAT pathways. This results in the expression of ISGs (summarized in Fig. 1b) [12, 32, 37, 38]. Type I and II IFN receptors are broadly expressed and thus most cell types respond to these cytokines, while type III IFNs are particularly relevant at anatomical barriers, such as the epithilia of the respiratory or gastrointestinal tracts [11, 12, 32].

Hundreds of IFN-regulated genes have been identified, many encoding proteins with antiviral activities [11, 12, 39–41]. Others desensitize the pathway to limit IFN signaling and to avoid toxic effects [42]. The precise control of IFN signaling is important as impairment results in defects in pathogen control [43, 44], while chronic activation of type I IFN signaling has been linked to autoimmune disease [45]. Among the ISGs are genes encoding members of the ART superfamily suggesting that ADP-ribosylation contributes to the antiviral response.

## Genes encoding ADP-ribosyltransferases are regulated by interferons

ARTs are enzymes capable of transferring ADP-ribose (ADPr) from NAD^+^ onto substrates. ADP-ribosylation comes in two forms, mono- and poly-ADP-ribosylation (MARylation and PARylation, respectively), which is catalyzed intracellularly predominantly by the ARTD family, including PARP and TNKS proteins (Box 1). ADP-ribosylation was discovered in the 1960s and numerous proteins have been identified as substrates. These are associated with many different biochemical and cellular functions, including DNA repair, viral replication, gene transcription and stress response [13]. Importantly, ADP-ribosylation is a fully reversible process. ADP-ribosylhydrolases contain either a macrodomain or a Ribosyl_crysJ1 domain capable of cleaving ADPr-ADPr or ADPr-amino acid glycosidic bonds (Box 2) [46]. Moreover, Nudix hydrolases cleave ADP-ribose to produce 5′ AMP and ribose-5-phosphate [47, 48]. Below we will focus on macrodomain-containing proteins as these have been identified in some viruses.

In addition to proteins, nucleic acids have been identified as substrates [49, 50]. Although a good part of the evidence comes from in vitro experiments so far, it is notable that enzymes in shellfish and in butterflies have been demonstrated to MARylate DNA, possibly as part of a defense mechanism [51–53]. Moreover, some toxins of bacterial toxin-antitoxin systems, which are involved in promoting persistence of cell populations, have been described as DNA ADP-ribosylating enzymes [54, 55]. It is tempting to speculate that ADP-ribosylation of viral nucleic acids might be of functional relevance.

An early observation linking ARTDs to IFN signaling was the demonstration that *PARP9* is responsive to IFNγ [56]. PARP9 was initially described as a risk factor in diffuse large B-cell lymphoma (DLBCL) and is upregulated in chemoresistant DLBCL [57, 58]. *PARP9* shares its promoter with *DTX3L* and, indeed, *DTX3L* is also IFNγ responsive. Both genes are activated in cells that express a dominant active form of STAT1 (Fig. 1b) [59]. Interestingly, PARP9 and DTX3L, a Deltex family member (see below), interact and participate in the activation of certain ISGs, suggesting a positive feedback loop [56, 60]. Another *PARP* gene identified as regulated by IFNs is *PARP13* [61, 62]. Moreover, in different experimental systems, including infection of human monocytes with *Borrelia burgdorferi*, spirochetes that promote an IFN response, the ISGs activated include *PARP10*, *PARP12* and *PARP14* [63–67]. Similar findings were obtained upon infection with for example a murine coronavirus (CoV) and with SARS-CoV-2 (for viral taxonomy see https://ictv.global/taxonomy/) [64, 68].

Comparing type I IFN-inducible genes between 10 different species, i.e. 9 mammals and chicken, revealed 62 up-regulated ISGs in all species and an additional 28 only in mammals, likely defining central components of the innate immune response [39]. These genes encode antiviral proteins, factors involved in PAMP sensing and in modulating IFN signaling, proteins implicated in antigen presentation, and several *PARP* genes (Table 1) [39]. The latter include the previously identified IFN-inducible genes, i.e. *PARP9*, *PARP10*, *PARP12*, *PARP13* and *PARP14*. These five genes were consistently activated, in many cases more than tenfold. In some species *PARP11* and *PARP15* were induced, while *PARP3*, *PARP4*, *PARP7*, *PARP8*, and *PARP16* were stimulated less efficiently (Table 1). Strikingly, these *PARP* genes encode mono-ARTs, except PARP13, which is thought to be catalytically inactive [69]. The genes encoding the PARylating enzymes PARP1, PARP2, TNKS1 and TNKS2 were not induced (Table 1 and Box 1). Together, these findings establish a link between innate immune signaling and the expression of a subset of *PARP* genes that encode MARylating enzymes.

**Table 1:**
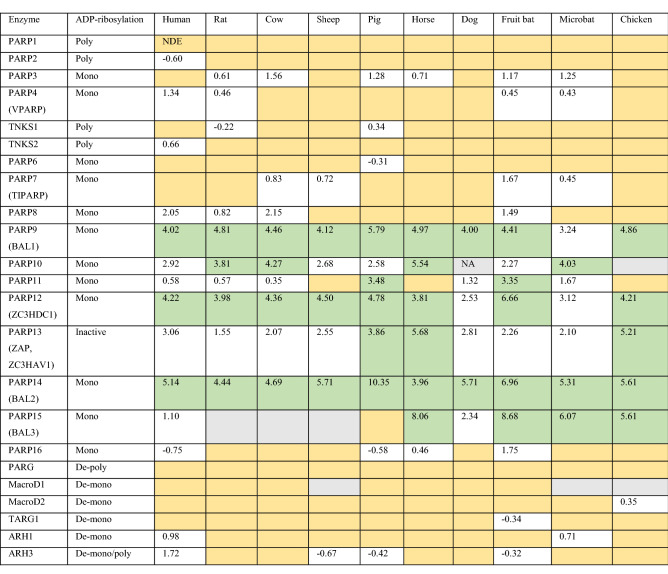
The expression of ARTD subfamily members, macrodomain-containing hydrolases, and ARH family members in response to interferon signaling is summarized

The data were obtained from http://isg.data.cvr.ac.uk [39]

The expression is indicated as log2 fold change. In green are genes whose expression is induced more than tenfold (log2 ≥ 3.33)

NDE/orange: not differentially expressed. This indicates genes that are neither induced nor repressed in a statistically significant manner

NA/grey: no data available

ADP-ribosylation: mono refers to ARTD enzymes capable of mono-ADP-ribosylating substrates; poly to ARTD enzymes synthesizing ADP-ribose polymer chains (iterative mono-ADP-ribosylation); De-poly and de-mono refer to enzymes that can degrade ADP-ribose polymer chains and substrate attached mono-ADP-ribose, respectively

In addition to IFNs, some *PARP* genes are also activated by PAMPs [70–73]. Whether these PAMP signals promote the expression of these *PARP* genes directly or whether these are indirect effects caused by activated IRFs driving IFN expression, thereby stimulating autocrine signaling, is not fully understood. In support of a direct effect are analyses of IRF1, a transcription factor directly activated by certain PAMPs [74]. IRF1 interferes with replication of some viruses and promotes the expression of *PARP9*, *PARP10*, *PARP12* and *PARP14* even in the absence of STAT1 [67].

Furthermore, it is of interest to mention that the different ADP-ribosylhydrolases capable of reversing ADP-ribosylation were also evaluated in the data set discussed above [39]. The genes encoding PARG and ARH3, both capable of hydrolyzing PAR chains, and the genes encoding MacroD1, MacroD2, TARG1, ARH1 and ARH3, which cleave the glycosidic bond between specific amino acid side chains and ADP-ribose, were not systematically regulated in response to type I IFN (Table 1 and Box 2).

Together these observations support the notion that MARylation contributes to innate immunity. Considering that genes encoding mono-ARTs are up-regulated upon IFN signaling, while genes encoding hydrolases are not, suggests that MARylation exerts predominantly antiviral effects. In support, some viruses encode a mono-ADP-ribosylhydrolase. However, this does not exclude that viruses may also exploit ADP-ribosylation for their propagation.

## ARTD protein expression and activation in innate immune signaling

While many reports demonstrate expression of *PARP* genes in response to PAMPs and IFNs, the expression and activity of the encoded proteins are less well studied. For PARP10, PARP12 and PARP14 enhanced protein expression in response to LPS has been documented [70, 71, 75]. Other PARPs have been more difficult to evaluate, mainly due to the lack of high quality reagents for protein detection and the low expression of some of these proteins. Even less clear is whether the catalytic activities of the different MARylating enzymes are altered in response to innate immune stimuli. While mass spectrometry studies define increasing numbers of substrates and ADP-ribosylation sites (see e.g. [76–82]), information that specifies enzyme–substrate pairs is still rare. Interestingly, a recent study suggests that ADP-ribosylation increases upon treatment with IFN as well as poly(I:C), a vRNA mimic [83]. This increase was unaffected by Olaparib, a potent PARP1/2 inhibitor, suggesting that PARP1 and PARP2 were unlikely to be involved. Whether the increase is due to activation of an ART or through inhibiting a hydrolase or both has not been clarified. Together, understanding the role of IFN-inducible PARPs in innate immunity will require the identification of substrates, to comprehend whether the relevant sites are differentially modified, and whether MARylation is of functional relevance.

## Evolution of mono-ARTs supports link to host–pathogen conflicts

*PARP* genes have undergone strong selection in primates [84]. In particular, it appears that *PARP13* is under positive selection, enhancing its antiviral activities [85]. Also *PARP9*, *PARP14* and *PARP15* seem to be under strong evolutionary selection [86]. The described rapid sequence adaptations are thought to reflect the arms race in host–pathogen conflicts. Of note is that the changes in sequence that were selected are particularly frequent in the catalytic domains, e.g. in PARP13 despite its apparent lack of catalytic activity, and in the macrodomains of PARP9, 14 and 15, domains linked to ADP-ribosylation-dependent signaling (Box 2). This supports the hypothesis that mono-ARTs are contributing to the antiviral innate immune response.

## PARP proteins in viral replication and propagation

The information depth regarding the consequences of mono-ARTs on viral replication and propagation is quite variable. Because mechanistic insight has only been obtained in some cases, the subsequent discussion is structured according to proposed mechanisms. Worth remembering is that PARP13 seems to be catalytically inactive as an ART and thus it does not teach us directly how ADP-ribosylation might interfere with viral replication. However, PARP13, which is also referred to as zinc finger (ZnF) antiviral protein (ZAP) or ZC3HAV1, is arguably the best studied PARP member with antiviral activity and it connects to other PARP enzymes, thereby likely affecting ADP-ribosylation (see below). Note that a lack of consequences on viral replication when single PARPs are manipulated should be interpreted with caution. The activity of a specific PARP may only unfold in cooperation with other ISG encoded proteins, including other IFN-inducible PARPs. Thus, while we learn more about individual PARPs and viral replication, a more complete picture will require to study cooperative activities of PARPs.

### Interference with viral replication

In an overexpression screen using Moloney murine leukemia virus (MMLV) as a model, PARP13 was identified as an inhibitor of viral propagation [87]. PARP13 is expressed as several isoforms. The most prominent are a long and a short version, referred to as PARP13 (PARP13.1/ZAPL) and PARP13.2 (ZAPS), respectively. Both encode all 4 ZnFs, but PARP13.2 lacks the pseudo-ART domain [88, 89]. The different isoforms have distinct activities in the innate immune response [88, 90]. PARP13 possesses robust antiviral activity in many experimental systems including, in addition to MMLV, certain alphaviruses, hepatitis B virus, influenza A virus, and SARS-CoV-2 [73, 85, 91–104]. Other PARPs, including PARP7, PARP9, PARP10, PARP12 and PARP14, also interfere with replication and propagation of some viruses [64, 66, 67, 105–110]. For PARP10 and 12 these effects are at least in part dependent on catalytic activity [66, 107, 108, 110]. Studying the effects of PARPs on different viruses reveals some selectivity, possibly due to different substrate specificities or the interaction with both host and viral proteins or nucleic acids (see below). For example, some viruses are not affected by PARP13, including yellow fever virus (YFV, *Flaviviridae* family) and Venezuelan equine encephalitis virus (VEEV, *Togaviridae* family) [95, 99, 108]. It is likely that PARP13 needs to cooperate with other IFN regulated gene products for antiviral activity [93, 111]. Similarly, PARP7 also shows some specificity, which may depend on the ability to interact with certain viral RNAs [105]. Thus, combinatorial effects of different PARPs and with other host factors will need to be considered.

### PARP proteins as sensors of viral RNA

**Fig. 2:**
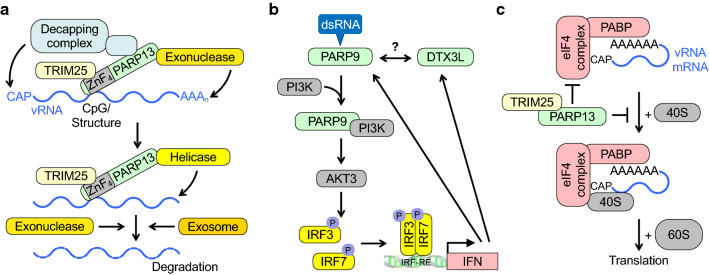
PARPs regulate the stability and the translation of viral and cellular RNA. **a** The Zinc fingers (ZnF) of PARP13 sense CpG-rich RNA, possibly in the context of secondary structure elements. CpG-rich RNA is typically a hallmark of non-self RNA. This is enhanced by TRIM25. PARP13 also recruits exonucleases, decapping enzymes, helicases and the exosome, which together promote RNA degradation. **b** PARP9 senses viral dsRNA and promotes the phosphorylation and activation of IRF3 and 7 through PI3K and AKT3 and subsequently the activation of *IFN* genes. This activation is not dependent on signaling complexes used typically by PRRs. These signaling events may function as a feed-forward loop. Whether this activity of PARP9 requires DTX3L is unclear. **c** PARP13 inhibits translation of certain cellular and viral RNAs by interfering with the eukaryotic initiation factors and the binding of the 40S ribosomal subunit to mRNA. The specificity of these effects is not fully understood

When analyzing the structural attributes of IFN-inducible PARPs, features that stand out are potential nucleic acid binding domains (ZnFs and RRMs) and macrodomains (Box 1). Indeed, several PARP proteins interact with viral RNA and thus may serve as PRRs. Again, PARP13 was the first identified. It interacts with vRNA through its ZnFs, which can stimulate RNA degradation (Fig. 2a). PARP13 seems to recognize multiple binding sites in both the 5′ or the 3′ untranslated regions (UTR) of MMLV, although no defined sequence was mapped [87, 112]. More recently, the CpG dinucleotide content was found to promote PARP13 binding. Synonymous mutagenesis of the HIV genome to increase the number of CpG dinucleotides resulted in sensitization to PARP13 [113]. The genomic positions of the additional CpG dinucleotides are relevant, suggesting that the sequence context is important [102, 114]. This is consistent with a high CpG content promoting an innate immune response [115–117].

PARP13 interacting factors are important for CpG dinucleotide dependent antiviral activity (Fig. 2a) [113, 118]. One is TRIM25, which appears to enhance RNA binding of PARP13 [119]. Because TRIM25 is involved in activating MAVS [1], promoting IFN expression might be a consequence of PARP13 sensing vRNA. Moreover, PARP13 interacts with the exosome, a structure that degrades RNA and is involved in immunity [120, 121]. Exosome-dependent degradation of RNA begins with removing the poly(A) tail. Indeed, PARP13 interacts with PARN, one of several exonucleases known to target poly(A), and indirectly with decapping factors [96]. In addition, evidence for the requirement of a DEAD box helicase that associates with PARP13 has been obtained [122]. Helicases are involved in resolving secondary structures of RNA, which is required for exosome-dependent degradation. These findings suggest that PARP13 bridges viral and possibly cellular RNAs (see below) to cellular complexes that unfold and degrade RNA. PARP7, similar to PARP13, uses its ZnF to bind to SINV vRNA and induces its degradation by recruiting the exosome [105].

PARP9 has also been suggested to function as a receptor of viral dsRNA. This interaction promotes the activation of IRF3 and IRF7 through the phosphoinositide 3-kinase (PI3K) and AKT3 pathway (Fig. 2b) [106]. IRF3 and IRF7 stimulate type I and III IFN gene expression, however, this appears to be independent of MAVS, suggesting that PARP9 functions as a non-canonical PAMP sensor. Consistent with this, *Parp9*^−/−^ mice are more susceptible to different viral infections [106]. Thus, evidence is accumulating that several PARPs function as PRRs, contributing to the first level of the antiviral response.

### Cellular RNA metabolism and protein translation

Many viruses affect the availability of cellular RNAs and control selective protein translation. Products of ISGs interfere with these processes [123]. Several PARP proteins have been suggested to alter protein translation. When expressed from a modified VEEV replicon, PARP7, 10 and 12 appear to strongly repress translation [109]. This might be due to synergistic effects with viral proteins, as these PARPs do not affect general translation in other cellular systems. As discussed above, vRNAs are regulated by PARP13. Moreover, it destabilizes certain cellular RNAs, for example the mRNA encoding TRAIL receptor 4 (TRAILR4), a decoy receptor [124]. Downregulation of TRAILR4 sensitizes cells to pro-apoptotic TRAIL signaling and thus may contribute to the antiviral function of PARP13. PARP13 was also found to inhibit translation at least in part by preventing the interaction of RNA with ribosomes (Fig. 2c) [125]. PARP13 interacts with eIF4A, a helicase important for initiation of translation [126]. The eIF4 complex is a frequent target of viral interference to promote translation of vRNAs [127]. In addition to stimulating RNA binding as described above, TRIM25 appears to participate in PARP13 mediated inhibition of viral translation [128]. Whether this is dependent on the E3 ligase activity of TRIM25 will need clarification. An indirect mechanism to control the availability of both cellular and viral RNAs is to interfere with Dicer/RNA-induced silencing complex (RISC), which contributes to antiviral immunity [129]. Subunits of RISC, including Ago2, are PARylated upon stress and damage-associated molecular patterns (DAMP) signaling [130, 131]. PARP13 interacts with Ago2 and interferes with miRNA function, possibly in complex with PARP12 and PARP15, offering additional mechanisms to control RNA stability and translation.

**Fig. 3:**
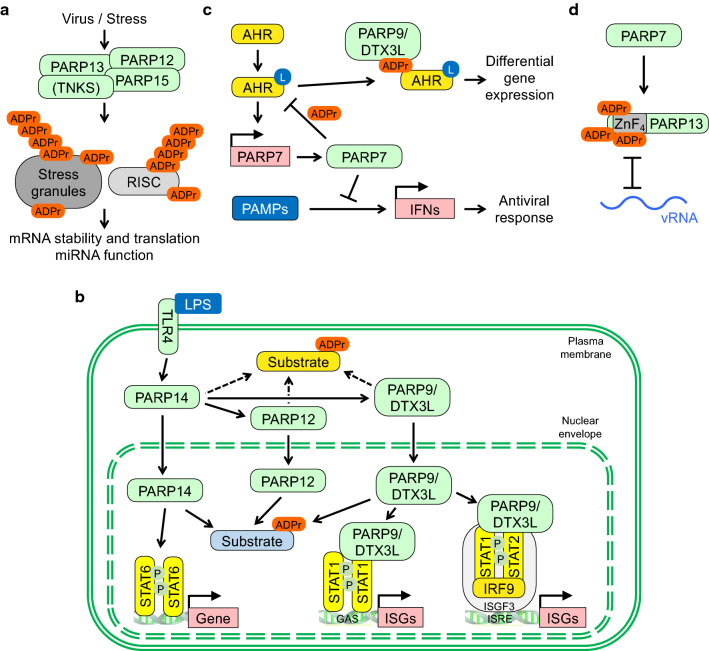
Cellular processes controlled by PARP networks. **a** Different forms of stress, including viral infection, stimulate stress granule (SG) assembly. Several PARPs are associated with SGs. G3BP1 and 2 are ADP-ribosylated and these two proteins are necessary for SG formation and for replication of certain viruses (see text). Moreover, components of the RNA-induced silencing complex (RISC) are substrates of PARPs. Both SGs and RISC are controlling the stability and availability of mRNAs, processes targeted by viruses. **b** The interactions of the indicated IFN-inducible PARPs are summarized. The activation of TLR4 by LPS promotes the nuclear translocation of PARP14 as well as PARP12 and PARP9/DTX3L. A shift in substrate ADP-ribosylation from the cytosol to the nuclear compartment is proposed. Moreover, PARP14 and PARP9/DTX3L have cofactor function and influence ISG expression. Not shown is the suggested antagonism of PARP14 and PARP9 in regulating STAT1 (see text). **c** Ligands (L) such as xenobiotics and microbial metabolites activate AHR, which stimulates *PARP7* expression. PARP7 in turn inhibits AHR function, at least in part through direct MARylation. MARylated AHR is read by PARP9 resulting in differential gene expression. PARP7 also modifies and inhibits TBK1 (not shown), a kinase activated by certain PAMPs, and thus prevents the expression of *IFN* genes. **d** PARP7 MARylates PARP13 at cysteines located in the ZnFs, which is suggested to interfere with the ability of PARP13 to sense CpG-rich vRNA

Many of the processes discussed above connect to stress granules (SGs), which are well-described to contribute to innate immunity [132–135]. Indeed, Ago2 and PARPs12-15 are found in SGs, pointing to an intimate relation of SGs with ADP-ribosylation [130, 136–138]. In support, ADP-ribosylation of both SG and RISC components are suggested to participate in antiviral activities (Fig. 3a) [136]. Because the effects on SG formation are thought to require PAR formation, the mono-ARTs PARP12, PARP14 and PARP15, and possibly others, may be responsible for initial MARylation. This might provide a seed for subsequent PAR formation, for example by TNKS1 or 2, which are also located in the cytosol and are associated with SGs when overexpressed [13, 130]. Together, a network of PARP proteins appears to contribute to SG formation, potentially regulating RNA stability and availability. Unraveling their precise interplay will be relevant to advance our understanding of the role of SGs for viral replication.

### Cellular interactors and substrates of IFN-inducible PARPs

A key question in understanding the role of PARPs in host–pathogen conflicts is to define their substrates. Both host and viral factors might be controlled by ADP-ribosylation. Different approaches have been employed including targeted approaches, protein arrays, chemical genetics approaches and BioID-based protein–protein interaction screens to define possible interactors and substrates. Because measuring MARylation in cells is cumbersome, many of the potential substrates await detailed analysis, including mapping modification sites to evaluate relevance.

The study of PARP10 and PARP14 on protein microarrays defined in vitro substrates [139], some of the PARP10 substrates could be verified in cells [140–142]. Through interaction and chemical genetic screens potential substrates for PARP10, PARP11 and PARP14 were identified [143, 144]. Interestingly, gene ontology (GO) analyses of these potential substrates suggest functions in signaling, metabolism and mRNA associated processes, among others, compatible with functions in innate immunity. One of the PARP14 substrates identified is PARP13, providing a link for an interaction network of PARP family members [144]. Other PARPs that are modified in cells include PARP9, PARP10 and PARP14 [145]. Support for a network is also provided by the interaction of PARP14 with MARylated PARP10 that is dependent on the ability of the macrodomains of PARP14 to read the modification [146].

Of note is that PARP14 translocates to the cell nucleus upon LPS stimulation, offering the possibility that nuclear substrates can be MARylated upon PAMP signaling, while cytosolic substrates may lose MARylation (Fig. 3b) [71]. These effects may be further augmented because the translocation of PARP14 also promotes the nuclear uptake of other proteins, including PARP12 and PARP9/DTX3L. Some PARPs affect transcription, for example PARP14 acts as a cofactor for STAT6 [147, 148]. Moreover, PARP9/DTX3L interacts with STAT1 and enhances its DNA binding on both ISRE and GAS elements and transcription of ISGs, suggesting a STAT1 cofactor function for PARP9/DTX3L (Fig. 3b) [59]. Consistent with this model is that the knockdown of either *PARP9* or *DTX3L*, which results in the repression of both proteins, abrogates the antiviral effect of active STAT1 [59]. The interplay of PARP14 and PARP9 has obtained further complexity when it was reported that STAT1 is MARylated by PARP14, which is antagonized by PARP9. This results in complex effects on STAT1 dependent gene transcription [149]. However, this study has provoked significant criticism [150], requiring further clarification about the interplay of PARP9 and PARP14. Also, a recent study did not find effects on ISG expression upon manipulating PARP9/DTX3L [83]. While LPS stimulates the nuclear translocation of mono-ARTs, LPS also stimulates PARylation through PARP1 and 2, which is inhibited by PARPi such as Olaparib [76, 151]. LPS has been described to induce DNA damage that activates PARP1 and PARP2 [152], and to promote PARylation of NFATc, a transcription factor involved in immune signaling [153]. Thus, under specific conditions, combination of MARylation and PARylation may affect gene transcription.

In addition to the above summarized accumulation of PARPs in the nucleus, PARP7 relocates to the cytosol upon stress to interact with vRNA and to stimulate mitochondrial damage [105]. Because BAX and BAK are involved, this may result in an apoptotic response. How general this process is remains to be determined. More recently, PARP7 substrates were found to be linked to gene transcription and microtubules, functions that might be relevant for virus-host interactions [154].

PARP9 was considered inactive. However, when interacting with DTX3L, this protein complex appears to have dual functions. In the presence of high NAD^+^ levels, the complex MARylates ubiquitin and blocks its use by E3 ligases. In contrast, when the complex is recruited to PAR chains through the PARP9 macrodomains, the E3 ligase activity of DTX3L is stimulated [60]. The authors suggested that PARP9 is responsible for this MARylation. However, a recent study finds that the RING-DTC domain of Deltex family E3 ubiquitin ligases such as DTX3L, independent of PARP9, is responsible for MARylation of ubiquitin [155]. It remains to be clarified whether PARP9 is indeed active or not. Recently, additional E3 ligases were identified in complex with PARP9/DTX3L as part of a newly forming protein module in myeloid cells upon LPS treatment [76]. These findings suggest an intimate relationship between PARP9/DTX3L and ubiquitination, a post-translational modification (PTM) prominent in controlling innate immune signaling [156].

Host proteins that have been linked to viral replication are the two highly homologous proteins RasGAP SH3-binding proteins 1 and 2 (G3BP1/2) [157]. These proteins are ADP-ribosylated, although the relevant enzymes have not been identified [158]. As discussed further below, these modifications are reversed by viral hydrolases. G3BPs are essential for SG assembly, which at least for some viruses is relevant for replication [159, 160]. The knockout (KO) of *G3BP1* and/or *G3BP2* affects alphavirus replication to different degrees [161]. SG condensates form through liquid–liquid phase separation (LLPS), suggested to be dependent on the RNA-binding capacity of G3BPs. PTMs modulate the ability of G3BPs to form condensates [162–164], and thus ADP-ribosylation of G3BP proteins might affect SG function and consequently viral replication.

The studies summarized in this section suggest that many of the interactors/substrates of IFN-inducible PARPs have antiviral activities. Although in many instances the full molecular consequences are not yet understood, as discussed further below, MARylation is moving into the focus of virus–host conflicts.

### Feedback response in interferon signaling

Deregulated IFN expression and/or signaling is associated with different diseases, including chronic viral infection or autoimmune diseases [45, 165–167]. For example, the lack of a proper response to IFNs due to inherent IFNAR1 deficiency results in adverse reactions to live virus vaccines [168]. More recently, an impaired IFN response has been found associated with severe COVID-19 [169, 170] (reviewed in [166, 171]). Unlike the antiviral activity of PARP13, PARP13.2 appears to be part of a negative feedback mechanism as it is involved in the degradation of several *IFN* mRNAs, thereby restraining the IFN response [90]. Others have reported that PARP13.2 promotes RIG-I signaling during the antiviral response, which enhances IFN production, and thus contributes to a positive feedback loop [172]. How these two opposing functions of PARP13.2 are regulated remains to be resolved.

PARP11 is a poorly studied family member that is induced by IFN and subsequently reduces IFN signaling. Mechanistically, PARP11 was shown to MARylate the ubiquitin E3 ligase β-transducin repeat-containing protein (β-TrCP), which promotes the poly-ubiquitination and degradation of IFNAR1 [173]. The consequence is reduced IFN signaling, contributing to the negative feedback response. Whether PARP11 also has antiviral activity has not been reported.

The aryl hydrocarbon receptor (AHR) is activated by ligands such as dioxin and other xenobiotics, but also microbial metabolites and thus functions as a PRR. Ligand binding promotes DNA binding and gene expression, one of its targets is *PARP7* [174–180]. PARP7 inhibits AHR at least in part by MARylating the receptor (Fig. 3c) [181–183]. Moreover, PARP7 induced by constitutively activated AHR interferes with PAMP signaling and, in accordance, IFN production is enhanced in AHR knockout cells, which hampers viral replication [184]. The PARP7 catalyzed MARylation of AHR is read by PARP9/DTX3L, which contributes to deregulated expression of a subset of AHR target genes [185]. Thus, the role of PARP7 in innate immunity is complex by limiting the host response but also by interfering with virus replication and propagation. Such a feedback mechanism might also be relevant during CoV infection, which promotes AHR activation by an unknown mechanism [186]. Moreover, this suggests that PARP9/DTX3L also possesses activating functions, for example through STAT1, but also repressing functions in combination with PARP7 MARylated AHR.

An additional level of feedback signaling by PARP7 has been described recently. PARP7 was found to MARylate proteins with functions in the immune system, including PARP13 (Fig. 3d) [187]. PARP7 has been reported to MARylate cysteines, unlike other IFN-inducible PARPs that modify glutamates and aspartates [181, 187]. PARP13 is MARylated by PARP7 at several of the cysteines that are responsible for coordinating Zn^2+^ ions, resulting most likely in inactivating the ZnFs and thus preventing RNA binding (Fig. 3d). This function may also be important to limit the innate immune response. So far, no eukaryotic hydrolase has been identified that reverses Cys-MARylation and thus the modification may provide a permanent inactivation of PARP13. Of note is the recent finding that a bacterial enzyme, SpyMacroD*,* which is part of a toxin-antitoxin system in *Streptococcus pyogenes*, can hydrolyze MAR-Cys (Box 2) [188]. This provides a first link to reversibility of cysteine MARylation. When discussing different acceptor sites, an additional mechanistic aspect is worth pointing out. While MAR and PAR chains can be distinguished by some readers, including macrodomains, the macrodomains of PARP9 may distinguish MARylation sites also according to the acceptor amino acid [13, 146, 189–191].

## The viral response to MARylation representing one arm of innate immunity

Viruses have developed many strategies to evade the innate immune response, including avoiding recognition by PAMP receptors and by interfering with IFN-dependent signaling [192, 193]. Because the available information suggests strongly that PARP mediated MARylation antagonizes viral replication and propagation, questions that are important to address include which strategies viruses developed to deal with MARylation and whether viral proteins are substrates. For both these questions we only have partial answers that we discuss in this section.

### Viral interference with cellular MARylation

Macrodomains, structural elements closely associated with ADP-ribose metabolism, are found in all kingdoms of life (Box 2) [46, 194–197]. Upon the realization that some viruses possess macrodomains, a potential role in antagonizing IFN-induced MARylation was hypothesized. Indeed, the viral macrodomains belonging to the alphavirus, orthohepevirus, alpha-CoV and beta-CoV genera have been identified as MAR-selective hydrolases [70, 198–203]. In some cases the relevance of macrodomains for replication has been documented [107, 199, 201, 204–207].

Beta-CoV, including SARS-CoV-2, possess a positive-sense, single-stranded RNA ((+)ssRNA) genome of roughly 30 kb [208–210]. SARS-CoV-2 encodes 16 non-structural proteins (nsPs), the corresponding information is encoded by the 5’ two-thirds of the viral genome. Additional open reading frames encode accessory and structural proteins. The nsP3 protein of SARS-CoV-2, a membrane bound multidomain protein, contains three macrodomains. Of these, only Mac1 binds ADPr [211–213]. Mac1 of SARS-CoV-2, SARS-CoV, Middle East respiratory syndrome coronavirus (MERS-CoV) and murine hepatitis virus (MHV, a CoV) functions as MAR hydrolase [202, 214]. These studies also demonstrate that Mac1 is important for efficient viral replication. In contrast, Mac2 and Mac3 do not possess hydrolase activity. Instead, they bind to nucleic acids or host proteins, thereby promoting replication [215–218]. An additional study supports the modulatory role of the catalytic macrodomain in MHV. General PARP inhibitors were able to interfere with viral replication only when an MHV strain was used, in which the nsP3 macrodomain was inactivated [64].

While in CoVs the macrodomain hydrolase activity enhances viral replication, the catalytic activity of the macrodomain of certain alphaviruses is necessary for replication [107, 199]. It is remarkable that of the four nsPs of alphaviruses, which typically have a (+)ssRNA genome of 10–12 kb, nsP3 contains a macrodomain with MAR hydrolase activity [219, 220]. The high selectivity of the macrodomains for MAR over PAR supports the notion that MARylating PARPs contribute to antiviral defense [70, 107].

As pointed out above, G3BP proteins are ADP-ribosylated. Early on it was found that SINV nsP3 is in complexes containing G3BP1 and G3BP2 [221]. The nsP3 proteins of other viruses were also found to colocalize with G3BP [222]. Of note is that several of the over 30 alphaviruses, which include a large number of mosquito-borne vertebrate pathogens [223, 224], possess two FGxF-like motifs in nsP3. This motif mimics a G3BP binding domain and may modulate G3BP function and SG assembly [220]. Of note for the discussion here is that the catalytic activity of the CHIKV nsP3 macrodomain was suggested to inhibit SG formation [158]. These findings, which need further verification, suggest that CHIKV nsP3 reorganizes G3BP condensate formation and thus may influence SGs.

**Fig. 4:**
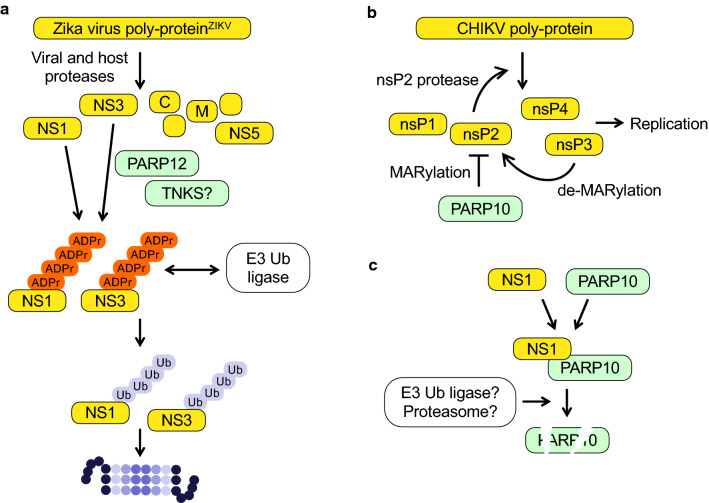
ADP-ribosylation of viral proteins and interaction with PARPs. **a** The non-structural proteins NS1 and NS2 of Zika virus are ADP-ribosylation substrates of PARP12 and potentially of a TNKS. The resulting PARylation promotes the recruitment of an E3 ubiquitin ligase. Subsequent poly-ubiquitination induces proteasomal degradation. At which point ADP-ribosylation is reversed has not been clarified. **b** The protease domain of the non-structural protein nsP2 of Chikungunya virus is MARylated by PARP10, which inhibits the proteolytic activity. As a result viral non-structural polyprotein processing and thus replication is inhibited. This is antagonized by the viral macrodomain of nsP3. **c** The non-structural protein NS1 of avian influenza virus interacts with PARP10 resulting in PARP10 degradation. Whether this involves poly-ubiquitination and proteasomal degradation has not been resolved

It is possible that viruses have developed additional means to deal with IFN-induced MARylation. In one study, it was suggested that the NS1 protein of avian influenza virus interacts with PARP10 and induces its degradation (Fig. 4c) [225]. In parallel, a cell cycle arrest was observed, although it is unclear whether this is the result of PARP10 loss or some other NS1 effect on the host. Nevertheless, PARP10 is antiviral in several experimental settings and thus is an attractive target for viral intervention.

### Viral ADP-ribosylation substrates

Recent studies have identified viral proteins as substrates of IFN-induced PARP enzymes. These include the nonstructural proteins NS1 and NS3 of Zika virus (ZIKV, *Flaviviridae* family) [110], the nucleocapsid proteins of several viruses [226], and the nsP2 protein of CHIKV [107]. PARP12 inhibits ZIKV replication by targeting NS1 and NS3. This results in PARylation dependent proteasomal degradation of the two viral proteins [110], which are generated upon processing of a polyprotein and are required for replication [227]. Because PARP12 is a mono-ART, the combined action of a MARylating and a PARylating enzyme, possibly TNKS1 or 2, is necessary. PAR chains can serve as binding sites for an ubiquitin E3 ligase, thereby promoting protein degradation (Fig. 4a) [13, 228]. The reported interaction of PARP12 with PAR chains, through one of its WWE domains [137], may be part of a positive feed-forward loop to promote efficient non-structural protein degradation.

The nucleocapsid proteins of several CoVs are ADP-ribosylated in cells [226]. Although the relevant PARP enzyme(s) has not been identified yet, it is of interest to note that plasmid encoded nucleocapsid was not modified. Instead, this ADP-ribosylation required infection with virus or replicon particles. Thus, it is possible that the relevant PARP(s) needs to be activated by viral infection. This might occur by directly binding to vRNA or by stimulating innate immune signaling. Alternatively, the subcellular localization might be critical and PARP and nucleocapsid are only in contact when additional viral proteins and/or the viral genome are present. It will be important to define the functional relevance of nucleocapsid ADP-ribosylation.

Processing of the non-structural polyprotein of alphaviruses is dependent on the protease encoded by nsP2, an important target for antiviral drugs [229, 230]. For CHIKV the protease is necessary for replication [107, 231]. PARP10 MARylates nsP2 and the isolated protease domain, which inhibits catalytic activity (Fig. 4b) [107]. The protease is reactivated when treated with the nsP3 macrodomain. Consistent with this finding is that a CHIKV replicon with an inactivated nsP3 macrodomain is replication defective.

Together, the findings discussed above provide the first few viral MARylation substrates that seem to be important for viral replication. It is likely that we are only seeing the proverbial tip of the iceberg.

## Viral macrodomains as targets for therapeutic intervention

The enzymatic activities encoded by viruses are important for replication, and some modulate the antiviral response executed by the innate immune system [192, 193]. Thus, targeting these activities with small molecules is of considerable importance to therapeutically interfere with viral replication and propagation [193, 208, 232–234]. The macrodomain hydrolases encoded by some RNA viruses, including SARS-CoV-2, CHIKV and Hepatitis E virus, are considered potential drug targets. Targeting viral macrodomains may be facilitated by the conserved fold of these domains and the large number of structures that are available [235].

Efforts have been undertaken to identify small molecule inhibitors of the catalytically active Mac1 of CoVs. Initial information, based on computational approaches and fragment screening, has been obtained that will be useful to develop Mac1 small molecule inhibitors [236–241]. Using homology to related macrodomains and to the PARG macrodomain resulted in two compounds that crystallize in the active site of Mac1 [238]. It will be interesting to see further biological studies of these compounds. In a large effort using crystallographic fragment screening and computational docking, many compound fragments were identified that interact with the active site of Mac1 [237]. Some of these fragment hits were further validated, providing a chemical framework as starting point for inhibitor development.

Also, efforts have been made to establish high throughput methods to screen for Mac1 inhibitors [242, 243]. In one study, 640 FDA approved compounds were tested that yielded a single substance, suramin [242]. However, further validation revealed low specificity. Suramin appears to have multiple effects as it also binds to the SARS-CoV-2 RNA polymerase [244]. Consistent with these findings, suramin has broad antiviral activities [245], and was reported to interfere with an early step of the SARS-CoV-2 replication cycle [246]. In a second study, dasatinib and dihydralazine were identified as Mac1 inhibitors [243]. Of note is that dasatinib, developed as an inhibitor of BCR-ABL and for the treatment of chronic myelogenous leukemia [247], did not inhibit MacroD2, the closest homolog of Mac1 present in humans.

A set of nucleosides and nucleotides were studied for binding to Mac1 using crystallography and modeling [240]. Interestingly, metabolites of remdesivir, including GS-441524, bound in the active center of Mac1. Because this metabolite inhibits both SARS-CoV-2 and MHV in cells and in a mouse model, respectively [248], GS-441524 may provide a hit compound for further development.

Specific inhibitors of viral macrodomains are not only relevant for CoVs but also for a number of alphaviruses that have a significant impact on the health of humans and other vertebrates [223, 249–251]. Several studies have used in silico strategies and high throughput screenings to identify compounds that target the CHIKV nsP3 macrodomain [252, 253]. Although it is unclear whether they are capable of inhibiting hydrolase activity, they showed weak activity in CHIKV infection models. Also flavonoids were identified as interactors of the CHIKV nsP3 macrodomain [254, 255], but again more detailed studies will be needed to verify the macrodomain as target. Finally, a recent study used compounds that were developed using in silico techniques to target MacroD1. Testing some of the compounds for their ability to interfere with Semliki Forest virus replication identified a compound with weak activity [256]. Together, these are promising studies that lay down the groundwork for developing much needed inhibitors of viral macrodomains.

## Outlook

The findings that multiple *PARP* genes but none of the genes that encode the antagonizing hydrolases are induced in response to IFNs suggest a broad antiviral role of MARylation. This is consistent with some viruses possessing macrodomains with de-MARylating activity. How do viruses that do not encode a catalytically active macrodomain deal with the PARP response? Because MARylation is widespread, it is well possible that such viruses have developed alternative strategies. One possibility is that de-MARylating activities are associated with as yet unidentified domains. These might be related to the ARH fold or be distinct from the presently known structures. It will be interesting to see whether indeed unidentified “hidden” hydrolases exist.

Other possibilities include that viral proteins affect the activities or the specificities of PARP enzymes, thereby diverting these proteins from critical host and/or viral substrates. The identification of HPF1 as a cofactor of PARP1, which changes its substrate specificity, has provided exemplary evidence for redirecting specificity of a PARP enzyme [257]. Not only may the interaction of viral proteins with PARPs interfere with their antiviral activities, as suggested for the avian influenza virus NS1—PARP10 interaction described above, we imagine that it may redirect PARPs from an inhibitory to a neutral or even a supporting activity for viral replication. Additionally, multiple viral effectors control PAMP and IFN signaling and thus may affect PARP expression and activation in ways that have not been elucidated yet. Together, the published findings delineate an important role of MARylation as an antiviral PTM.

Despite these notions, we would like to point out the possibility that some pathogens, including viruses, do not interact with mono-ARTs and MARylation. Consequently, these pathogens may not require activities counteracting MARylation and thus they may lack MAR hydrolases or regulators of mono-ARTs.

Key to understanding the MARylation arm of innate immunity and its interaction with viruses will be to define more broadly relevant substrates, both viral and host factors. With the progress in the development of mass spectrometry techniques and detection tools to study MARylation in cells, we expect to see a boost in MARylation substrates. These will be interesting to study regarding virus–host interactions. Moreover, mapping modification sites and understanding the functional consequences of MARylation both on host as well as on viral proteins will be instrumental to define the role of MARylation as an antiviral mechanism, but also how viruses might capitalize on this PTM for their own advantage.

Beyond proteins, with the detection of modification of nucleic acids by some of the IFN-inducible PARPs [49, 50], the question becomes obvious whether this is a modification that occurs on viral genomes and whether this is part of the innate immune response. If so, the functional consequences need to be addressed as well as the activities of viral macrodomains on nucleic acid substrates.

Finally, defining relevant substrates as well as viral effectors will provide opportunities to develop small molecule inhibitors for therapeutic approaches. As many of the viral proteins possess multiple functions, developing inhibitors, or more generally binders, further to proteolysis targeting chimera or PROTACs might also be relevant for future therapeutic strategies, as already noteworthy in cancer therapy [258, 259]. The development of small molecule inhibitors/degraders of viral macrodomains is certainly of high importance as multiple studies have demonstrated the importance of the de-MARylating activity. Thus, such inhibitors may have high therapeutic value. We are optimistic that the current efforts, particularly on SARS-CoV-2, will help to develop viral macrodomain specific therapeutics.

Box 1 Brief summary of eukaryotic, intracellular ADP-ribosyltransferases and their catalytic activities ADP-ribosylation is a fully reversible modification of proteins, nucleic acids or metabolites (see Box 2 for the description of hydrolases). The reaction involves the transfer of ADP-ribose (ADPr) from the redox cofactor β-nicotinamide adenine dinucleotide (NAD^+^) onto the respective substrate [13, 49, 262]. It links signaling to basic cellular metabolism due to the central role of NAD^+^ in e.g. glycolysis and the Krebs cycle [263]. The main representatives of intracellular ADP-ribosyltransferases (ARTs) belong to the ARTD (ARTs of the diphtheria toxin-like) family [14], which share a highly conserved catalytic domain that resembles the ART domain of diphtheria toxin [264]. Besides, ARTDs have diverse additional functional domains linking them to numerous cellular processes. The enzymes are named PARP or TNKS. Some of these are capable of synthesizing ADPr polymers by iteratively transferring ADPr units. However, most enzymes transfer a single unit of ADPr (summarized in the figure). For PARP13 no catalytic activity has been reported so far, whereas findings with PARP9 are controversial (see the text). Several of the genes encoding ARTDs are interferon responsive and thus belong to ISGs. Various ADPr acceptor amino acids are under debate, including glutamate, aspartate, serine, arginine and cysteine. Of note is that the selectivity or the activity of a PARP protein may depend on cofactors. For example, the activity of PARP1, the target of clinically relevant inhibitors, shifts from modifying acidic amino acids to serine in the presence of HPF1 [265, 266].
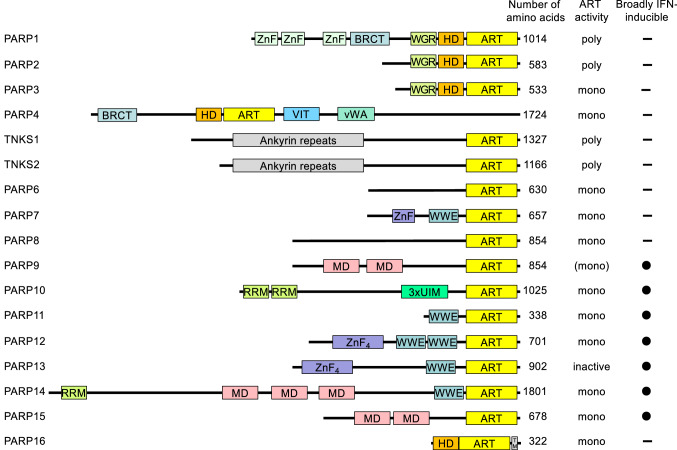

The domain architecture of ARTD family members is summarized: ART, ADP-ribosyltransferase domain; BRCT, BRCA1 C-terminus domain; HD, helical domain; MD, macrodomain; RRM, RNA-recognition motif; SAM, sterile alpha motif; TM, transmembrane motif; UIM, ubiquitin-interaction motif; vWA, von Willebrand factor type A domain; VIT, vault protein inter-α-trypsin domain; WGR, conserved Trp-Gly-Arg motif domain; WWE, three conserved residues Trp-Trp-Glu motif domain; ZnF, Zinc finger.

Box 2 ADP-ribosylhydrolases reverse ADP-ribosylation Both mono- and poly-ADP-ribosylation are reversible modifications, however, no eukaryotic hydrolases have been identified that remove mono-ADP-ribosylation from cysteines and lysines. Several proteins with hydrolase activity have been described in higher eukaryotes. One group contains macrodomains. These domains are closely associated with NAD^+^ metabolism. Many macrodomains are capable of interacting with free ADPr and/or with ADPr-modified substrates and thus may function in disseminating the information carried by substrate ADP-ribosylation. Other macrodomains function as hydrolases and reverse ADP-ribosylation [46, 197]. The latter is true for MacroD1, MacroD2 and TARG1, which function as mono-ADP-ribosylhydrolases. PARG is a fourth protein with a macrodomain fold providing hydrolase activity. It is expressed as multiple splice variants that localize to either the nucleus or the cytosol. PARG possesses poly-ADP-ribosylhydrolase activity but is unable to cleave the bond between ADPr and an amino acid side chain. A second group of proteins referred to as ADP-ribosyl-acceptor hydrolases or ADP-ribosyl-glycohydrolases (ARH1-3) possesses homology to a family of selenoproteins termed SelJ [267]. ARH proteins share a common Ribosyl_crysJ1 domain [46].

The ability to read and potentially regulate ADP-ribosylation is conserved among all kingdoms of life, as hydrolases are also found among bacteria and some RNA viruses. Macrodomain hydrolases associated with microorganisms seem to be relevant in stress response, some of which represent one part of bacterial toxin-antitoxin systems [54]. Several RNA viruses, including alphaviruses and Coronaviruses, possess macrodomains that function as mono-ADP-ribosylhydrolases [46]. They are important for viral replication and host immune modulation, as discussed in the text. These hydrolases appear to target MARylated acidic amino acids. Recently, SpyMacroD, a bacterial hydrolase of a toxin-antitoxin system in *Streptococcus pyogenes* [268], was shown to remove MAR from cysteine [188]. The Ribosyl_crysJ1 domain has also been identified in proteins of some microorganisms. For example, the domain is found in proteins associated with toxin-antitoxin systems that function in interbacterial competition [269].
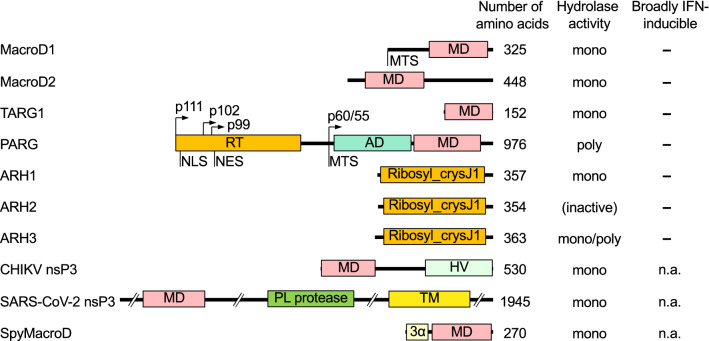

The domain architecture of selected members of the above-mentioned ADP-ribosylhydrolases is summarized: AD, accessory domain promoting MD function; HV, hypervariable domain; MD, macrodomain; MTS, mitochondrial targeting sequence; NES, nuclear export sequence; NLS, nuclear localization sequence; nsP, non-structural protein; PL protease, papain-like protease domain (cleaves the viral non-structural polyprotein); Ribosyl_crysJ1, ADP-ribosylation/Crystallin J1 fold; RT, regulatory and targeting domain; TM, transmembrane; 3α, 3-helix bundle (3 α-helices that coordinate a Zn^2+^ binding loop). The different isoforms of PARG are indicated.

## Data Availability

Not applicable.
